# The Need for Nurse Interventions in Sex Education in Adolescents

**DOI:** 10.3390/ijerph18020492

**Published:** 2021-01-09

**Authors:** Ľuboslava Pavelová, Alexandra Archalousová, Zuzana Slezáková, Dana Zrubcová, Andrea Solgajová, Zuzana Spáčilová, Erika Krištofová, Alica Slamková

**Affiliations:** 1 Department of Nursing, Faculty of Social Sciences and Health Care, Constantine the Philosopher University in Nitra, 949 74 Nitra, Slovakia; aarchalousova@ukf.sk (A.A.); dzrubcova@ukf.sk (D.Z.); asolgajova@ukf.sk (A.S.); zspacilova@ukf.sk (Z.S.); ekristofova@ukf.sk (E.K.); aslamkova@ukf.sk (A.S.); 2 Faculty of Nursing and Professional Health Studies, Slovak Medical University, 833 03 Bratislava, Slovakia; zuzana.slezakova@szu.sk

**Keywords:** adolescent, school nurses, sexual health education, Slovakia

## Abstract

*Background:* Developmentally appropriate evidence-based sexual health education should be included as part of a comprehensive school health education program and be accessible to all students. The registered school nurse is a valuable resource to parents and educators in this area and supports the implementation of evidence-based sexual health education programs that promote healthy sexual development for adolescents. *Methods:* The research group consisted of 438 adolescents aged 12 to 15 years in a selected region in Slovakia, 186 boys and 252 girls. Average age of the girls was 13.2 and the boys 13.3 years. A nurse—a specialist in community nursing—collected the data using a self-designed questionnaire. The questions evaluated by the five-point Likert scale focused on finding out the knowledge and attitudes of adolescents to the role of school nurses regarding sexuality and reproductive health. Results were analyzed using parametric comparison tests with significance value 0.05: Student *t*-test for independent samples. *Results:* The girls and the boys most often drew information on sexuality and reproductive health from their parents and friends. The evaluation of the adolescents’ views on who should be a competent professional in the field of sexual education at schools found statistically significant differences between the boys and girls. For the boys and girls, a sexologist received the most significant assessment of competence. The interest in a school nurse in a school environment would be statistically significantly more appreciated by the girls compared to the boys, not just for solving problems related to healthy lifestyle, but also regarding sexuality, parenting and marriage. The adolescents consider the education for marriage and parenthood as the least discussed issue at present. In evaluating topics the adolescents would discuss, there were statistically significant differences between the boys and girls. *Conclusions:* A community or school nurse would also be able to successfully perform sexual education at schools. In Slovakia, this applied nursing discipline is lacking.

## 1. Introduction

Adolescence is defined as the time between puberty and adult independence, which depends on individual development and culture [1]. Adolescence begins with the onset of physiologically normal puberty and ends when adult identity and behavior are accepted. This period of development corresponds roughly to the period between the ages of 10 and 19 years, which is consistent with the World Health Organization’s (WHO’s) definition of adolescence [2]. Adolescence is a time of change: changes to hormones and the body, changes in the social environment, and changes to the brain and the mind. Although most young people develop into healthy adults, adolescence confers vulnerability to mental health problems [1]. Puberty is related to sexual maturation. Changes which take place in the bodies of boys and girls modify their different attitudes towards their surroundings, themselves and sexuality. The formation of gender identity to one’s own sexuality depends on primary socialization within the family, the influence of peers and friends, as well as the educational system [3]. Puberty changes differ in both the sexes. On an average, females experience these changes a few months earlier than males [4]. Early maturing boys having good body image are more confident, secure and independent as compared to late maturing boys. However, they may have increased aggressiveness due to a surge of hormones. They are more likely to be sexually active and participate in risky behavior [5,6]. Early maturing girls are very self-conscious and insecure [7]. They are more likely to face sexual advances from older boys, have more chances of unwanted pregnancies and are more likely to be exposed to alcohol and drug abuse. The boys as compared to girls are more likely to face negative health outcomes like aggressive behavior and depression [8]. During adolescence, the girls are more likely to face a higher risk of negative health outcomes; girls are more vulnerable to adverse outcomes [9].

In Slovakia, the number of young people aged 10–19 is 542,491, which represents 5.25% of the total population. Adolescents form the most sensitive social group, which has been hit hard by social changes in society [10]. Adolescence is a complex developmental stage in which changes that promote the transition from childhood to adulthood occur in the physical, psychological and social spheres [11]. Children and adolescents need accurate and comprehensive education about sexuality to practice healthy sexual behavior as adults. Early, exploitative or risky sexual activity may lead to health and social problems, such as unintended pregnancy, including HIV and AIDS [12]. However, patterns in the sexual behavior of adolescents and young adults in Central and Eastern Europe seem to be changing. A decrease in the age at which they become sexually active is evident, particularly among females, leading to a narrowing of the gap between boys and girls regarding the time of sexual initiation [13]. Risky sexual behaviors among youths and adolescents are a global public health issue [14]. In Slovakia, sexual behavior of children is risky and the level of education in the field of sexuality is significantly lower than in developed countries. The authors point to a lower (to no) level of communication about sexuality in families. Lack of knowledge is replaced by communication among peers. Sexually active young people in Slovakia consider the risks and talk about the risks of pregnancy to a lesser extent. They also use a condom and contraception less frequently. Sexual coercion (women coerced by men) is more common among Slovak men and Slovak women, as there are more problems with setting borders and controlling sexual events [15]. One of the factors that contribute to lower risk behavior (smoking, drinking, early sexual behavior) is the school environment and belonging to it. School belonging is affected not only by the individual but also by peers, families and teachers; the broader organizational school system; and social values, norms and policies [16]. In countries where compulsory sex education is introduced, there is a later start to sex life, a lower incidence of abortions and teenage pregnancies, as well as a lower incidence of sexually transmitted infections [17]. Adolescents control their healthy lifestyle by reason only to a certain extent. Adolescents often unknowingly prefer a risky lifestyle that helps them to solve problems associated with adolescence, such as low self-esteem and the search for their identity. Education for a healthy lifestyle should be based primarily on the syndrome of risky behavior during adolescence. Risk behavior syndrome includes both psychosocial and sexual risk behaviors [10].

Comprehensive sexual health education (SHE) reduces risky sexual behavior and increases protective behavior in adolescents. It is important to understand how professionals responsible for implementing SHE policy interpret state and local policies and what influences their commitment to formal SHE policy implementation [18]. A community or school nurse would also be able to successfully perform sexual education at schools. Unlike many countries in Europe and the USA, where school nursing is also enshrined in law, this applied nursing discipline is lacking in Slovakia. In these countries, school nursing focuses on the prevention and control of communicable diseases, health counseling, health promotion, ensuring and monitoring a safe and beneficial environment, health management and others [19,20]. A school nurse, as a community care professional working in schools, is a person who contributes to the improvement of children’s mental health and plays an important role in prevention and in sexual education and health [21].

In schools, the number of children who need an individual approach from teachers, specific adaptation of the educational environment or specialized help from experts during compulsory school attendance is increasing from year to year. Students with disabilities, with various chronic health issues and risky behaviors are becoming a common phenomenon at schools. Schools perceive this as a problem they cannot overcome. Teachers are not allowed to administer medicines because they do not have the necessary competencies to perform healthcare tasks. Schools do not have other professional staff available to perform these tasks instead of them [20]. The solution in these situations could be the introduction of nurses into the school environment. In addition to direct medical care in working with children, school nurses would assist in the preparation of prevention programs and healthy lifestyles, including sex education and preparation for parenthood. School nurses would perform routine health examinations in schools on each child, followed by monitoring of their health. They would coordinate the cooperation of school management, teachers, paediatricians, psychologists and, if necessary, social and community workers [20]. 

Slovakia has an obligation under international United Nations (UN) conventions to ensure the conditions for the education of all children. The school and healthcare systems lack professionals in the field of community nursing care with a focus on the school environment and care of students’ health. In 2007, the Government of the Slovak Republic approved material on the procedures for implementing the WHO European Strategy on Health and Healthy Development of Children and Adolescents, where it points to the priorities and intersectoral cooperation between departments in promoting health and healthy development of children and adolescents. The designated working group was to propose a Health Care Concept in the field of general care for children and adolescents. One of the requirements was the reintroduction of the school health service with a recommendation to entrust it to university-educated nurses [22].

The aim of health promotion is to get young people to take care of their health. Health promotion deals with activities that aim to inform society about possible ways and alternatives for disease prevention, improve the knowledge of individuals and motivate them to change the wrong way of life. The European Convention on Human Rights and European Parliament have issued a resolution on sexual and reproductive health and rights [23]. The UN Convention on the Rights of the Child calls for and defends the need for children and young people to have access to objective holistic sex education [24]. The resolution emphasizes the differences in the sexuality needs of adolescents and adults. The rights of young people, their opinions and attitudes, and competence are essential for the development and implementation of sex education health programs with subsequent validation. Their active participation, in cooperation with parents, teachers and other professionals and volunteers, is important [23,25]. Adherence to the principles of sex education is important, considering the specific sensitivity of boys and girls and different lifestyles [26]. It promotes a holistic and positive approach to sex education, considering psychosocial and biomedical aspects [27]. The open discussion on sex in Slovakia is still largely taboo and research in this area is still relatively unsatisfactory. Former studies were previously focused on HIV infection and sexual behavior. Adolescent sex education has changed. The European Commission states that girls and boys should be free to express their opinions and feelings and to pursue their chosen educational and professional paths without restrictive standards [3]. 

This study was focused on understanding the current knowledge and attitudes of adolescents towards a school nurse. It maps interventions of health education focused on sex education in the environment of primary schools for a group of adolescents. It finds out the differences in knowledge, identification and needs of adolescents in sexual education in the school environment in a selected region in Slovakia. 

## 2. Materials and Methods 

### 2.1. Sample and Procedure

The research group consisted of 438 adolescents aged 12 to 15 who attended primary schools in southern Slovakia with a willingness to cooperate, with the consent of school management and parents. Participants were selected using convenience sampling. There were 186 boys and 252 girls. Average age of the girls was 13.2 and the boys 13.3 years (SD = 1.2, resp. 1.1). The data were collected using a self-designed questionnaire, which comprised of 23 questions. The questions were evaluated by the Likert scale (1 max–5 min). Scores were approximately normally distributed; therefore, parametric tests were used for all the conducted comparisons. Before the data were collected, content validity of the questions was evaluated by two experts. Pilot evaluation of the questionnaire was also conducted on a small sample of students (*n* = 10). No further changes in the questionnaire were needed. The data collection was carried out and the knowledge was assessed by a nurse specialist in community nursing. The questions were focused on finding out the knowledge and attitudes of adolescents to the role of a school nurse, identification of basic theoretical knowledge in the field of reproductive health and sexuality, and mapping the implementation of health and sexual education of adolescents in the school environment in a selected region of Slovakia.

### 2.2. Statistical Analysis

The statistical analysis was performed using the SPSS 22.0 software (IBM, Armonk, NY, USA). *t*-test for independent samples was used for comparisons of boys and girls in all variables. Means and standard deviations were used as summary statistics. Normality of the variables was evaluated with the Kolmogorov–Smirnov test (variables were statistically non-significant) and using Skewness and Kurtosis descriptive characteristics (all indices were <1.0).

#### Ethical Statement

The study was approved by the management schools in region Nitra (1295–2020). Parents were informed about the study via school administration and could opt out if they disagreed with their child’s participation. Participation in the study was fully voluntary and anonymous, with no explicit incentives provided for participation.

## 3. Results

The results of the research are presented in Table 1, Table 2, Table 3, Table 4, Table 5 and Table 6. We assumed that most of the adolescents addressed had information in the field of health, marriage, parenthood and sex education from parents. In the first question (Q-1), the adolescents answered from whom and to what extent they obtained information in this area, and in the second question (Q-2), whom would they consider the most important person for a conversation about sexuality. 

From the group of boys, the largest source of information was parents (arithmetic mean, M = 1.08). It was the same with the girls (M = 1.04). No statistically significant differences were found between the girls and boys in other responses to sources of information. They had no information at all from a sexologist and volunteers (M = 5.00). They had no information from a school nurse (this position of a nurse in the healthcare system in Slovakia is not established). The boys (M = 1.65) considered friends and parents (M = 1.75) as the most suitable people regarding sex education. The girls responded similarly. There were no statistically significant differences between the responses with respect to gender. An interesting finding is the shift in the preference of a school nurse in boys (M = 3.02) and in girls (M = 3.52).

The evaluation of the adolescents’ views on who should be a competent professional in the field of sex education at schools found statistically significant differences between the boys and girls. For the boys and girls, a sexologist received the most significant assessment of competence. Compared to the boys, the girls also considered a teacher (*p* = 0.063), a sexologist (*p* = 0.006) and a practitioner (*p* < 0.001) more competent. 

The interviewed adolescents were interested in establishing the position of a school nurse in the environment of primary schools for the purpose of sex education and parenting education. The interest in a school nurse would be statistically significantly more welcomed by the girls compared to the boys (*p* < 0.001). The importance of a school nurse in relation to a student (M = 2.01), as well as the importance in relation to the teacher (M = 2.32), was also evaluated higher by the girls where at the same time statistically significant differences in these evaluations were found between the boys and girls. 

If a school nurse worked at school, both boys and girls would welcome addressing problems of a healthy lifestyle. Statistically significant differences with respect to gender were found in three areas: a general solution to the issue with discussion (*p* = 0.005), parenting education (<0.001) and the topic of marriage (<0.001). All three areas would be more appreciated by girls.

No statistically significant differences were found in evaluation of current education at school according to gender. The boys rated the issue of nutrition and healthy diet the most (M = 1.98) and the issue of education for marriage and parenthood the least (M = 4.66). The girls mentioned active movement the most in the evaluation of current education at schools (M = 1.89) and the least, the same as the boys, the issue of education for marriage and parenthood (M = 4.89). 

Statistical differences between the boys and girls were found in the evaluation of topics on which adolescents would lead discussions. The topics of a responsible approach to sex life, sexual abuse, parenting, contraception and gender equality would be more appreciated in the discussions by the girls. Compared to the girls, the boys would like to discuss sex life more. 

## 4. Discussion

It is the position of the National Association of School Nurses (NASN) that developmentally appropriate evidence-based sexual health education should be included as part of a comprehensive school health education program and be accessible to all students. NASN recognizes the role of parents and families as the primary source of education about sexual health. 

The registered professional school nurse (hereinafter referred to as school nurse) is a valuable resource to parents and educators in this area and supports the implementation of evidence-based sexual health education programs that promote healthy sexual development for all students [28]. Sex education for adolescents in the conditions of the Slovak Republic does not yet have a separate subject. It is only a part of school subjects of ethical education, biology, civics or organizational classes. Curricula are not compulsory. The topic of sexual behavior is in the curriculum of the cross-sectional subject education for marriage and parenthood [29]. It also deals with a healthy lifestyle to encourage adolescents to start a family and have children in the future. Schools have a sex education coordinator who organizes thematic events or discussions with doctors, psychologists or other professionals. Compulsory subjects such as biology and civics may include topics like contraception, prevention of sexually transmitted diseases, health and psychological risks of changing sexual partners. The research team found [30] that a number of children with risky behavior and health problems do not have adequate care and conditions to meet their needs during their stay at school. They perceive the introduction of a school nurse into practice as the most appropriate solution. This is not a new idea, but an innovation of a proven system from the past. In the past before 1989, nurses worked in district outpatient clinics for children and adolescents, providing comprehensive health care, as well as prevention and education at schools. They find the current situation unsatisfactory. 

In Slovakia, the position of a school nurse, whose job description could also be sex education, is not established. We found that the girls and boys had the most common information about sexuality from their parents and friends. They did not often receive information from teachers, but in the future, they would be interested in talking with teachers in this area as well. The authors of the study [31] reported that teachers and the mass media were identified as the two most important sources of adolescents’ knowledge of sexuality. Parents were the primary source of knowledge on less taboo topics and general practitioners on HIV and AIDS topics. Peers and the mass media were important sources of information for sexually active adolescents, in contrast to adolescents who had no sexual experience and whose important sources of information were teachers and parents. In the evaluation of adolescents’ opinions on who should be a competent professional in the field of sex education in schools, the girls compared to the boys consider a teacher, a sexologist and a general practitioner more competent. The interest in establishing the position of a school nurse in the environment of primary schools for the purpose of sex education and parenting education would be more welcomed by the girls compared to the boys. The sample group included physicians and directors of kindergartens and elementary schools in Slovakia and documents concerning the school healthcare service. The results of questionnaires and interviews demonstrated a positive attitude to the position of the school nurse and implementation of her position in practice [32]. The research team found out that the founders of kindergartens, primary and secondary schools in towns and municipalities were interested in establishing a new position of a nurse at schools [33].

The authors [34] found positive interest in visits and discussions with nurses on sexual health, especially in boys. In the study carried out by the authors [35], they confirmed the greater trust of students towards the abilities of a nurse at school in dealing with issues such as the use of condoms than of teachers in health education. Nurses can use their unique combination of knowledge and skills to make a positive impact on adolescent sexual and reproductive outcomes. Nurses have the capacity and opportunity to disseminate information about sexual and reproductive health to adolescents and their parents in communities, schools, public health clinics and acute care settings [36]. We found out that the girls wanted to talk the most about sexual abuse and a responsible approach to sex life. Boys would discuss sex life the most. Significantly more girls than boys wanted to address gender differences and equality in the discussion. If a school nurse worked at school, both boys and girls would mainly appreciate solving problems concerning a healthy lifestyle. With a school nurse, the girls would also address the issue of sexuality, parenting education and the topic of marriage. In the study, the authors [37] found out in sixth grade students that the most common topics for discussion were sexual activity, female anatomy, reproduction and puberty. There were fewer questions about sexually transmitted diseases, sexual violence, drug use or alcohol. Students who participated in sessions that were taught by school nurses were more likely to report significant and sustainable changes in a broad range of sex-related cognitive mediators, including self-efficacy, condom-related beliefs and peer behavior beliefs, while those taught by health education teachers reported long-term impact on condom knowledge only [35]. Part of the research was a subjective assessment of the level of knowledge by adolescents. Adolescents subjectively marked the level of their knowledge in the field of sexuality and sex life as average. In five checking questions focused on the knowledge of the concepts of homosexuality, contraception, monogamy, sexually transmitted diseases and sexual abstinence, the boys answered correctly in 42.3% and girls in 51.13%. At least 93% of students in all schools said their level of knowledge was medium or high. Students in the school with the highest overall level of knowledge of human reproduction and sexuality had lower overall levels of sexual activity [38]. Results demonstrate that school-based sex education is an effective strategy for reducing HIV-related risk. Students who received school-based sex education interventions had significantly greater HIV knowledge, self-efficacy related to refusing sex or condom use, fewer sexual partners and less initiation of first sex during follow up [39]. We found out that adolescents mentioned the issue of education for marriage and parenthood as the least discussed topic at schools. Differences were also found in specific topics that the boys and girls would like to discuss in the field of sex education. Topics such as responsible approach to sex life, sexual abuse, parenting, contraception and gender equality would be more interesting for the girls. Compared to the girls, the boys would like to discuss sex life more. The majority of studies reviewed, which examined findings by gender, found no difference in perception of sexual health education by gender [40]. 

Sex education in the school curricula is a dynamic change for both health care and education in the countries. Involvement of the recipient is necessary for the program to be relevant and appropriate. There is still a need for a concerted effort from all stakeholders to take responsibility for sexuality education and not relegate it to be the prerogative of school teachers [41].

## 5. Conclusions

Nurses care for adolescents in a variety of settings, including communities, schools and public health and acute care clinics, which afford them many opportunities to improve adolescents’ sexual and reproductive health and reduce the rates of unplanned pregnancy and sexually transmitted infections [35]. The results are relevant to educators and healthcare providers who ask and answer questions from early adolescents regarding sexual health. The involvement of nurses in schools in Slovakia in the education of sex education and reproductive health is an ideal recommendation of this study. The results point to a significant interest of adolescents in the involvement of a nurse, a health worker, in education that would support the acquisition of knowledge and reduce risky behavior in the sexual field. In designing health programs for adolescents, the family should be involved in sex education. 

The limitations of this study are as follows: only a small group of adolescents participated in this study, and we did not find out the opinions of their parents, so we consider the findings to be partial. Another limitation of the study is the type of sampling, which was a deliberate, non-probable selection. The generalizability of the results is weaker. Further research is also needed to identify the attitudes and opinions of parents and teachers for more comprehensive findings.

## Figures and Tables

**Table 1:** Sources of information on sex education in adolescents.

Sources	Q-1			Q-2		
	Boys (M)	Girls (M)	*p*	Boys (M)	Girls (M)	*p*
Teacher	2.44	2.81	0.069	2.22	2.17	0.730
Non-teaching staff	2.23	2.33	0.427	2.66	2.48	0.215
School nurse	5.00	5.00	1.000	3.02	3.52	0.085
Psychologist	4.08	4.14	0.585	3.68	3.16	0.083
Sexologist	5.00	5.00	1.000	4.58	4.26	0.099
Volunteer	5.00	5.00	1.000	4.14	4.03	0.301
Parent	1.08	1.04	0.408	1.75	1.98	0.113
Friend	1.99	1.87	0.099	1.65	1.65	1.000
Other/who?	2.97	2.34	0.063	3.21	2.77	0.107

Explanatory notes: Q-1—question 1; Q-2—question 2; M—arithmetic mean; *p*—value of statistical significance.

**Table 2:** Competent professional in sex education for adolescents.

Competent Professional	Boys—M	Girls—M	*p*
Teacher	3.31	3.04	0.063
Non-teaching school staff	2.62	2.81	0.191
School nurse	2.08	2.12	0.750
Social worker	2.81	2.76	0.730
Psychologist	2.81	2.54	0.063
Sexologist	2.10	1.75	0.006
Volunteer	3.16	3.12	0.783
General practitioner	2.39	1.87	<0.001

Explanatory notes: M—arithmetic mean; *p*—value of statistical significance.

**Table 3:** Interest in a school nurse and the importance of her relationship with other people at school.

Interest/Importance	Boys—M	Girls—M	*p*
Interest in “school” nurse	2.59	1.61	<0.001
Student–nurse relationship	2.88	2.01	<0.001
Teacher–nurse relationship	2.74	2.32	0.004
Parent–nurse relationship	3.64	3.72	0.581
General practitioner–nurse relationship	3.68	3.88	0.169

**Table 4:** Areas of interest of adolescents in school education implemented by school nurse.

Areas of Interest	Boys—M	Girls—M	*p*
Healthy lifestyle	1.62	1.52	0.389
General solution of the issue with discussion	2.22	1.81	0.005
Responsible approach to sex life	2.36	2.23	0.370
Sexual abuse	2.99	3.24	0.085
Sex life	2.23	1.96	0.063
Sexual tolerance	2.15	2.39	0.099
Parenting education	3.34	2.11	<0.001
Marriage	3.42	2.15	<0.001

Explanatory notes: M—arithmetic mean; *p*—value of statistical significance.

**Table 5:** Evaluation of topics/areas of current education in schools.

Topics/Areas	Boys—M	Girls—M	*p*
Health education	2.23	2.28	0.667
Nutrition and healthy diet, drinking regime	1.98	1.99	0.945
Active movement	2.11	1.89	0.130
Sex education and sex life	4.59	4.66	0.630
Education to marriage and parenthood	4.66	4.89	0.113
Preventive activities	3.01	3.10	0.535
Health programs	2.31	2.23	0.581
Other (specify)	3.45	3.98	0.068

Explanatory notes: M—arithmetic mean; *p*—value of statistical significance.

**Table 6:** Topics that adolescents want to talk about and discuss at school.

Topics	Boys—M	Girls—M	*p*
Responsible approach to sexual life	3.33	1.91	<0.001
Sexual abuse	3.38	1.53	<0.001
Sex life	2.05	3.10	<0.001
Sexual tolerance	2.40	2.16	0.099
Education about parenthood	3.99	2.01	<0.001
Contraception	3.86	3.11	<0.001
Gender equality	3.99	1.68	<0.001
Other	3.55	3.69	0.469

Explanatory notes: M—arithmetic mean; *p*—value of statistical significance.

## Data Availability

Data sharing is not applicable to this article.
